# Velocimetry of GHz elastic surface waves in quartz and fused silica based on full-field imaging of pump–probe reflectometry

**DOI:** 10.1016/j.pacs.2024.100627

**Published:** 2024-06-11

**Authors:** Ruben Burger, Goran E. Hallum, Ramon Auer, Dennis Schweiger, David Redka, Matthias Domke, Christian U. Grosse, Heinz P. Huber, Datong Wu

**Affiliations:** a Munich University of Applied Sciences HM, Lothstr. 34, Munich, 80335, Bavaria, Germany; b Technical University of Munich - Chair of Nondestructive Testing, Franz-Langinger-Str. 10, Munich, 81245, Bavaria, Germany; c Vorarlberg University of Applied Sciences - Josef Ressel Center for Material Processing with Ultrashort Pulsed Lasers, Feldgut 9, Rankweil, 6830, Vorarlberg, Austria

**Keywords:** Surface wave imaging, Photoacoustics, Pump–probe technique, Anisotropic media, Velocimetry

## Abstract

This study reports an imaging method for gigahertz surface acoustic waves in transparent layers using infrared subpicosecond laser pulses in the ablation regime and an optical pump–probe technique. The reflectivity modulations due to the photoelastic effect of generated multimodal surface acoustic waves were imaged by an sCMOS camera illuminated by the time-delayed, frequency-doubled probe pulses. Moving the delay time between 6.0nsto11.5ns, image stacks of wave field propagation were created.

Two representative samples were investigated: wafers of isotropic fused silica and anisotropic x-cut quartz. Rayleigh (SAW) and longitudinal dominant high-velocity pseudo-surface acoustic wave (HVPSAW) modes could be observed and tracked along a circular grid around the excitation center, allowing the extraction of angular profiles of the propagation velocity. In quartz, the folding of a PSAW was observed. A finite element simulation was developed to predict the measurement results. The simulation and measurement were in good agreement with a relative error of 2 % to 5 %.

These results show the potential for fast and full-field imaging of laser-generated ultrasonic surface wave modes, which can be utilized for the characterization of thin transparent samples such as semiconductor wafers or optical crystals in the gigahertz frequency range.

## Highlights

• Pump–probe technique for visualization of GHz surface waves in transparent media.
• Fast, full-field detection of HVPSAW and SAW modes in quartz and fused silica wafers.
• Measurement of direction-dependent propagation velocities of SAW and HVPSAW.
• The experimental results were validated with FEM simulations (discrepancy 2 % to 5 %).
• Two-temperature hydrodynamic simulation (1D) of the excited pressure amplitudes.

## Graphical abstract

**Figure:**
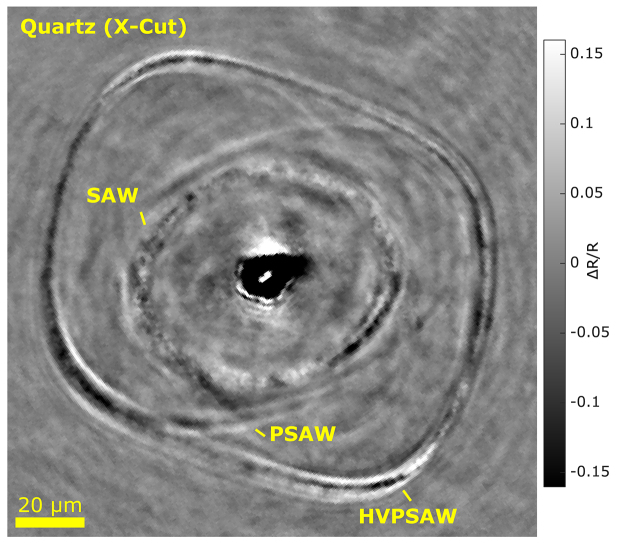

## 1 Introduction

Analysis of the propagation of high-frequency acoustic surface waves on crystalline media based on imaging of the wave field, i.e., the spatial and temporal progression of the wave front, has potential in nondestructive evaluation and material characterization. Since wave propagation is directly coupled to elastic parameters, measurement of wave velocities can be used to determine the stiffness tensor of the material. Knowledge of these parameters is important for crystalline materials due to their various applications in nonlinear optics [1] or as a basis for surface acoustic wave filters in the gigahertz range [2] for analog signal conditioning used in current mobile communication devices. Current methods for determining the material parameters of layers in the GHz range include Brillouin spectroscopy [3] that requires scanning at multiple propagation angles to obtain angular velocity information or resonant ultrasound spectroscopy [4] that analyses the vibrational eigenmodes of well-defined sample shapes.

Popularized by Thomsen et al. [5] for detection of phonons on the surface of thin films excited by picosecond laser pulses, various techniques that utilize pump–probe microscopy to image acoustic wave propagation in transparent media following strong laser-matter interaction and nonlinear absorption have been developed [6]: In addition to shadowgraphic pump–probe measurements of pressure waves in bulk materials [7], [8], [9], [10], [11], [12] and shock wave visualization in liquid layers [13], [14], surface acoustic waves (SAW) can be imaged by pump–probe microscopy at normal incidence [15]. Domke et al. [16] have performed pump–probe microscopy measurements in hard tissues by resolving the change in the speckle signal induced by acoustic wave propagation. Wäger et al. [17] have shown the propagation of acoustic waves on a glass surface in combination with shock waves in the air layer.

The group around Prof. Wright [18], [19], [20], [21], [22], [23] have performed extensive pump–probe experiments on 2D imaging of surface acoustic waves on transparent samples with a metallic coating layer in the nanometer range. Here, both Rayleigh/surface acoustic waves (SAW) and high-velocity pseudo-SAW (HVPSAW) were observed using optical pump–probe techniques below the ablation threshold of the metallic coating. Detection is carried out point-wise, mainly interferometrically, by detecting the phase change caused by out-of-plane movement of the interface due to the passing surface waves [15].

A less common detection method is the use of the photoelastic effect [20]. In-plane and out-of-plane motions may be observed by a local change in the electrical susceptibility of the material as a result of photoelasticity. This results in local variations of the partial reflectance coefficient at the interface, leading to a contrast in reflectivity, which can be detected using a photodiode [21] or CCD cameras [13], [14], [24]. While [13], [14] visualize laser-induced shock wave propagation in liquids, in [24], nonlinear acoustic propagation of bulk waves arriving at a surface layer has been observed in the femtosecond regime in gold. Laser-generated longitudinal pressure pulses in a thin gold layer, originating behind the measurement plane, are detected on the sample surface by reflectometry. Here, the picosecond delay in arrival times of the pulse on the surface due to intensity-dependent nonlinear propagation velocities could be detected.

With cameras, fast imaging can be achieved at the cost of reduced sensitivity. For both interferometric methods as well as photoelastic detection schemes carried out with a photodiode in the reviewed literature, the imaging is achieved by moving the measurement spot across a defined measurement area at fixed time delays using a two-axis stage system that requires long measurement times for the generation of one image.

The aim of this work was to employ a fast, full-field imaging mode for surface waves in transparent solids, enabling quantitative measurement of wave propagation in all directions of the surface plane. This alternative method was realized by measuring the reflectivity change due to the photoelastic effect using an sCMOS camera instead of a single-point photodiode. Except for very low illumination conditions, sCMOS sensors have shown comparable performance to CCD sensors in various scientific applications [25], [26], [27]. Due to reduced sensitivity of full-field measurements, the method requires ablative excitation to achieve sufficient signal strength. Although surface waves were observable without averaging, we used N=10 averages for noise reduction. Our pump–probe setup uses a common path to focus both the pump and probe beam and thus requires access only to one side of the sample.

Based on the work of Wäger et al. [17], which showed the feasibility of instantaneous imaging of surface wave fields using such a common path setup, our goal was to apply the method to anisotropic media and to perform a quantitative analysis of wave propagation. The angular velocity profile of the SAW and HVPSAW was measured by tracking the wave front evolution over time. Finite element simulations were developed to verify the origin of the experimental results.

## 2 Theoretical background

The wave propagation in elastic media is governed by the general equation of motion given by the equality of internal stress force density and reaction of the mass per unit volume:

(1) $$\rho \cdot {\overset{¨}{u}}_{i}=\frac{\partial \sigma_{ik}}{\partial x_{k}}$$

with density ρ [$kg\;m^{-3}$], particle displacement vector $u_{i}$ [m], stress tensor $\sigma_{ik}$ [Pa] and coordinate axis $x_{k}$ [m] using Einstein summation convention [28]. The coupling between stress and strain is – linearity assumed – given by the generalized Hooke’s law:

(2) $$\sigma_{ik}=C_{iklm}\cdot \frac{\partial u_{m}}{\partial x_{l}}$$

with strain tensor ${ɛ}_{lm}=\partial u_{m}/\partial x_{l}$ [29]. The fourth-order stiffness tensor $C_{iklm}$ [Pa] contains material parameters and couples stress with strain. The number of independent elements in $C_{iklm}$ depends on the symmetry of the material and can range from 21 for triclinic symmetry to only two for isotropy [30]. In the case of trigonal symmetry, as in the quartz crystal investigated in this work, six elements of the stiffness tensor are independent [31].

Substitution of (2) in (1) leads to the system of equations governing the elastic wave propagation in anisotropic media:

(3) $$\rho \cdot {\overset{¨}{u}}_{i}=C_{iklm}\cdot \frac{\partial^{2}u_{m}}{\partial x_{k}\partial x_{l}}\;.$$

Various strategies for solving (3) can be found in the literature [32]. Here, we will briefly discuss the results regarding interface waves in both materials investigated in this work.

For isotropic media, bulk waves have parallel (longitudinal wave) or orthogonal (shear wave) polarization to the propagation direction with distinct propagation velocities. On a free surface, the well-known Rayleigh or SAW mode appears, which has a propagation velocity slightly below the bulk shear velocity and elliptical, mainly out-of-plane, particle movement. The SAW mode is confined to the surface with a penetration depth of approximately one wavelength and thus has low energy losses as propagation is limited to one spatial plane. A longitudinal dominant HVPSAW mode can be detected on the surface that propagates with velocities close to the longitudinal bulk mode [31], [33]. For the isotropic case, this mode is also termed leaky SAW (LSAW) [34]. Canfield et al. [34] and Royer and Valier-Brasier [31] provide an overview regarding the naming conventions of surface wave modes. To avoid confusion, the notation for the anisotropic case (see below) is used universally in this work. The reach of HVPSAW is limited compared to that of the SAW due to energy leakage in the form of radiation of a shear wave in the bulk material [35]. Due to isotropy, all modes propagate without angle dependence.

For anisotropic media, even bulk wave solutions are generally not polarized parallel to or orthogonal to the propagation vector. Here, they are classified into one quasi-longitudinal (QL) and two quasi-transversal ($QT_{V/H}$) modes by their dominant polarization component with three distinct propagation velocities [31]. For surface waves, the equivalent of the SAW mode in the isotropic case also exists, as well as the HVPSAW, although both have direction-dependent parameters. In addition, for certain directions and crystal orientations, more solutions are possible. For example, a pseudo SAW (PSAW) mode can appear with velocities slightly below the fastest transverse bulk wave [31]. Some of these special modes exhibit low propagation attenuation and find application in high-frequency SAW filter devices [36]. In anisotropic cases, depending on the curvature of the phase velocity profile, directions with multiple solutions for the group/energy velocity can exist, leading to characteristic cusp shapes in the wavefronts [30].

## 3 Materials and methods

### 3.1 Experimental setup

**Fig. 1:**
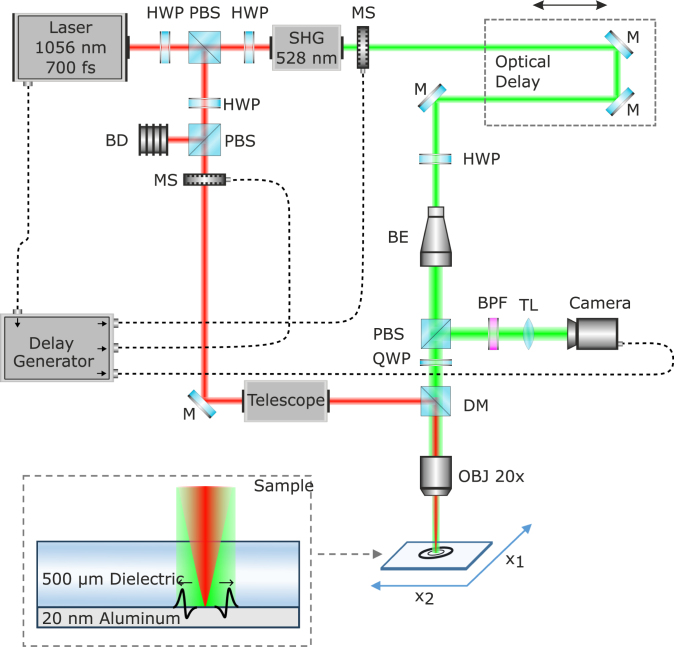
Pump–probe reflectometry setup (similar to [37]) and detail of microscope setup. Components: half-wave plate (HWP), polarizing beam splitter (PBS), second-harmonic generator (SHG), mechanical shutter (MS), mirror (M), beam expander (BE), dichroic mirror (DM), quarter-wave plate (QWP), objective (OBJ), band-pass filter (BPF), tube lens (TL), beam dump (BD).

The pump–probe setup (Fig. 1) used to image the elastic waves was similar to that published in [37] using only a mechano-optical delay line to resolve the delay time of the probe pulse. The 700fs, 1056nm laser pulse generated by an Nd:glass source with a repetition rate of 500Hz is split using a polarizing beam splitter (PBS) into pump and probe arms. The energy of the pump beam is then adjusted by using a motorized half-wave plate (HWP) and a second PBS with the excess energy absorbed by a beam dump (BD). Fast mechanical shutters (MS) are synchronized with the laser pulses using a delay generator to ensure that only one pulse is transmitted in each measurement in both the pump and the probe arm. The beam passes through an adjustable telescope for spot size adjustment before being combined with the probe beam path in a dichroic mirror (DM) and focused (diameter at $e^{-2}$ intensity ≈20µm) onto the dielectric-Al interface by a 20x objective (OBJ, Mitotoyo M Plan Apo 20). For the probe arm, the laser pulse is frequency-doubled to 528nm using a second-harmonic generator (SHG). With a mechanical delay stage, the pump–probe time delay can be adjusted between 6nsto11.5ns. Before the probe beam is expanded and recollimated with a beam expander (BE), a rotatable HWP in combination with the PBS allows the probe beam power adjustment. A quarter-wave plate (QWP) circularizes the previously linear polarized probe beam before illuminating the sample through the objective. The depth of focus of the objective was 1.6µm. The reflected probe beam after the QWP is linearly polarized orthogonal to the incoming beam and is directed to the camera by the PBS. A band-pass filter (BPF) ensures that only the probe wavelength reaches the camera. A tube lens (TL) projects the image of the sample on the camera sensor (pco.edge 4.2.LT) [38], which has a resolution of 2048 pixels by 2048 pixels with a full well capacity of 30000 $e^{-}$. The intra-scene dynamic range is up to 91.5dB. The quantum efficiency for the probe wavelength is 80% with a typical readout noise of 1.3 $e^{-}$. The camera is able to detect changes in the reflectivity of ${\frac{\Delta R}{R}}_{min}\approx 3\times 10^{-5}$, which is well within the expected signal amplitude [24].

Since quartz exhibits uniaxial birefringence and the optical axis of the material (z axis of the material frame) is normal to the probe pulse propagation for the x-cut quartz sample, the polarization state of the probe pulse is altered crossing the material twice since the sample basically acts as a high-order waveplate ($d_{sample}\approx 1900\lambda_{probe}$). This can lead to reduced image brightness and thereby reduced sensitivity compared to non-birefringent samples with identical experimental settings, when the polarization state of the reflected pulse is oriented towards the transmission direction of the PBS. This can be compensated for by rotating the HWP above the BE until the intensity of the reflected orthogonal components is sufficiently high. This compensation can only account for static birefringence; strain-induced birefringence from acoustic waves can still influence the sensitivity of the setup. During the measurements with birefringent samples, we often observed a second, weaker excitation spot caused by the split in the pump beam when the prob e beam polarization was not aligned with the optical axis of the sample.

During each measurement, 56 ablation events were recorded from the initial delay of 6.0ns to 11.5ns with increments of 0.1ns. Between shots, the sample was moved 250µm laterally using a motorized stage to an area not affected by previously ablated areas. Two images were recorded: one image several milliseconds before the pump pulse was sent, $R_{bef}$, and one image at the time offset between the pump and the probe pulse defined by the length of the optical delay line, $R_{dur}$. Here, $R_{xxx}$ denotes a two-dimensional array of intensity values returned by the camera sensor. A full measurement cycle had a duration of approximately four minutes for 56 pump–probe delays (≈4s per delay). Each measurement was repeated ten times for SNR improvement leading to a total measurement time of 10×4min=40min. Pump pulse energies were measured at the focal point and are kept at 4.6µJ (peak fluence approximately $1.4\;J\;{cm}^{-2}$) for both investigated samples. The intensity of the probe pulse is adjusted so that the images are as bright as possible without saturating the camera sensor during the measurement. The energy of the probe pulse was around 1.5µJ, measured after the objective. For full-field illumination, the beam waist was larger than the field of view of 665µm. Using the measured probe pulse energy, this gives an upper limit for the probe pulse fluence of $0.8\;mJ\;{cm}^{-2}$.

### 3.2 Sample preparation

Two samples were selected for this study: SiO_2_ in the form of fused silica and x-cut quartz. Fused silica was selected as an isotropic reference material because of its comparable simple elastic wave propagation. Quartz was selected because of its anisotropy that arises from its trigonal crystal symmetry. Since quartz also exhibits piezoelectricity, the possible influence on wave propagation was checked in advance. Using a modified version of the *christoffel* Python toolset [39] the energy velocities of the three bulk wave solutions were calculated in the yz-plane of quartz with and without the piezoelectric effect. The $QT_{H}$ and QL modes are unaffected; only the $QT_{V}$ mode is slightly modified due to piezoelectricity. The maximum electromechanic coupling coefficient K defined as [31]

(4) $$K=\sqrt{\frac{v_{P}^{2}-v^{2}}{v_{P}^{2}}}$$

with v ($v_{P}$) as the bulk velocity without (with) the piezo effect is approximately 0.14 as is also reported in [40]. The maximum change in velocity is in the range of 1%. The Supplemental Materials contain the calculated angular distribution of K for x-cut quartz. Lewis and Patterson [41] could not detect an influence on the SAW velocities of x-cut quartz due to piezoelectric coupling. The piezoelectricity of quartz is not considered further in this work but has to be considered when investigating samples with high piezoelectric coupling coefficients.

Both samples were obtained as optical grade wafers with a diameter of 2 inches and a thickness of 500µm. The surfaces finish was of optical quality with a wavefront error smaller than λ/10 over the beam diameter. This allowed focusing through the sample and provided a smooth surface for wave propagation.

The samples were coated with a 20nm thick layer of aluminum on one side by physical vapor deposition. The chosen layer thickness is large enough for a sufficient reflectivity of the probe wavelength of 528nm (R≈0.84) without detectable influence on the propagation of acoustic waves in the observed frequency range. The smallest resolvable acoustical wavelengths are at least one magnitude larger than the aluminum layer. Assuming that 10 pixels per wavelength are required to accurately resolve the wavefront, the smallest wavelength is around $\lambda_{min}=10\cdot d_{px}=3\;µm$ with $d_{px}$ being the pixel pitch of the sensor adjusted for the magnification of the setup. For a HVPSAW (SAW) mode with a velocity of $6000\;m\;s^{-1}$ ($3000\;m\;s^{-1}$) this translates into a maximum frequency of 2GHz (1GHz). The lower frequency limit is determined by the slower SAW and thus by the thickness of the sample, since the penetration depth is approximately one wavelength [42]. At a thickness of 500µm, using a safety factor of 10⋅λ and a propagation velocity of $3000\;m\;s^{-1}$, the lower frequency limit will be in the 60MHz range.

### 3.3 Experimental data analysis

The raw images were first averaged 10 times to improve SNR. A section $A_{norm}$ outside the ablation area was selected as a reference. For each time delay, the reference reflectivity $R_{ref}$ was calculated from both images by

(5) $$R_{ref}=\frac{1}{2\;n_{A}}\left(\sum R_{bef}\left(A_{norm}\right)+\sum R_{dur}\left(A_{norm}\right)\right)$$

with the number of pixels in the reference area $n_{A}$ ($n_{A}\approx 2000$ pixels). The relative change in reflectivity ΔR/R was then calculated by dividing the difference in pixel value by the sum of the averaged pixel values in the reference area [24]

(6) $$\frac{\Delta R}{R}=\frac{R_{dur}-R_{bef}}{R_{ref}}\;.$$

**Fig. 2:**
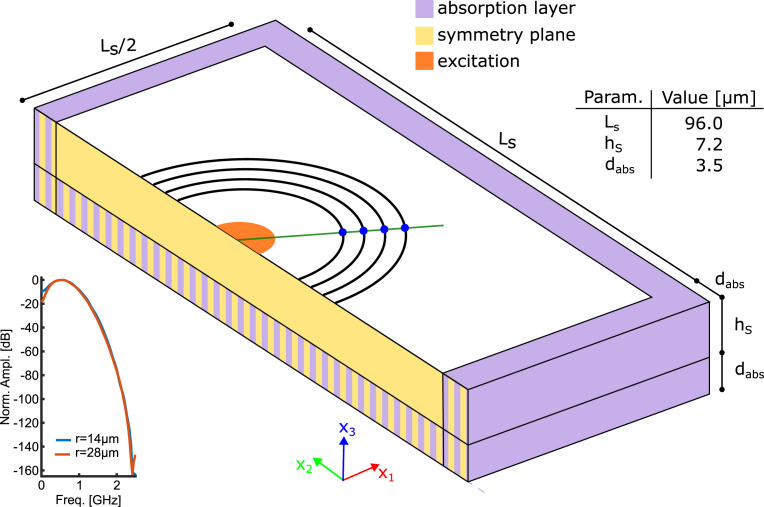
Setup for 3D elastic wave simulation in COMSOL. The elastic waves were excited at the top (orange semidisk) and recorded using a grid with cylinder symmetry (blue points). Symmetry planes (yellow) and absorbing layers (purple) were used to reduce the simulation size. The green line shows the measurement method to follow the propagation of the modes along a specific direction. The inset table gives the values of the parameters used. The Lab/Comsol coordinate frame is shown at the bottom. Inset shows the frequency spectrum of the $x_{3}$-components of the propagating wave in fused silica for two distances from the center. (For interpretation of the references to color in this figure legend, the reader is referred to the web version of this article.)

To smooth the images, a 2 by 2 pixel median filter was applied on each image. The image stacks were then analyzed using a custom Matlab application that allows manual recording of peak locations along an angle from the center of excitation (see Fig. 2; screenshot in the Supplemental Materials). Repetition of this peak determination for several delay times allowed the propagation of the wave to be traced. For both datasets, wavefront positions were recorded at six time delays per direction with 5∘ increments for each mode, resulting in 432 data points. The pixel distance from the marked peaks to the center was translated to µm by multiplying with the known pixel pitch $d_{px}=325\;nm$. A linear regression of the peak positions and times was performed to estimate the propagation velocity.

### 3.4 Simulation of wave propagation

To predict the measurement wave propagation at the interface was simulated using the *elastic waves* module of COMSOL Multiphysics 5.5, which uses the discontinuous Galerkin method to solve time-explicit wave propagation [43]. The simulation assumes linear elasticity and can therefore not model nonlinear effects of the wave propagation or the formation of shock waves. Since the anisotropy of the surface waves was of interest in this work, a full 3D simulation of the samples was necessary to observe all propagation modes. In the COMSOL version used in this work, piezoelectricity is not available in the material model. The simulation is purely mechanical without considering the optical or thermal physics and therefore does not model the ablation process itself.

The simulation setup is shown in Fig. 2. The excitation spot is located in the center of the sample (orange semidisk). The elastic wave was induced by a pressure pulse normal to the sample surface to match the excitation in the ablation regime. The excitation was Gaussian in time and with a rectangular spatial profile. The duration of the simulation excitation pulse was two magnitudes longer (σ=200ps; bandwidth 2.5GHz) than the duration of the experimental optical pulse. This was to reduce the simulation complexity, and thereby the computational requirements. The pulse length of the simulation was different from the optical experimental pulse duration and was chosen so that the generated frequency spectrum is comparable to the observable spectrum of the measurement system, which is mainly limited by the camera resolution. Since only the surface waves were of interest here, the simulated volume was kept as small as possible. This required two considerations: First, the cross section ($L_{s}=96\;µm$) of the geometry was halved using the symmetry in the $x_{2}x_{3}$ plane (yellow area) without losing information. Second, the thickness of the sample was set as low as possible without disturbing the SAW propagation in the desired frequency window ($h_{s}=7.2\;µm$). To emulate a continuing solid, reflections from the bottom interface were damped using absorbing volumes (thickness $d_{abs}=3.5\;µm$) and low-reflecting boundaries at the bottom $x_{1}x_{2}$ plane. The same combination was also utilized on all remaining sides of the model to minimize reflections. The chosen absorption setting reduces the reflections by a factor of 100 to 1000.

The simulation volume was discretized with fourth-order tetraedric Lagrange elements with a spacing of 1.2µm. The time range was 0nsto9ns after excitation. The simulation time step (3.5ps) is chosen by the solver based on the mesh size and predicted propagation speeds of the elastic wave. However, mesh discretization further limits the propagating frequency range. The inset in Fig. 2 shows the frequency spectrum of the $x_{3}$-components of the propagating elastic waves at two distances from the excitation center. The −6dB range is 200 MHz to 950 MHz with a center peak at 500 MHz. An attenuation of low-frequency components due to the bottom absorption layer is observed. This could be prevented by increasing the simulation thickness; however, this would require more powerful hardware. Reflection of the waves from the bottom interface is not observed, which prevents dispersive Lamb wave modes from forming. The validity of the SAW regime was tested in a separate parameter study and can still be confirmed for even thinner simulated thicknesses. The simulation contained $165\times 10^{6}$ degrees of freedom and the runtime was 35 h. The discretization parameters were validated for convergence in advance. To reduce the amount of generated data, only surface data was recorded.

The measurement method is sensitive to changes in the permittivity of the material due to the photoelastic effect. However, the simulation did not take into account the photoelasticity or optical properties and only solved the stress and strain tensors, as well as the particle velocity and pressure. Therefore, the simulation data was normalized since the simulated amplitudes cannot be correlated with the observed change in reflectivity $\frac{\Delta R}{R}$. However, since both simulated and observed quantities were coupled to the propagation of elastic wave modes, the propagation velocities were correlated, which is important for the verification of the measurement data.

**Fig. 3:**
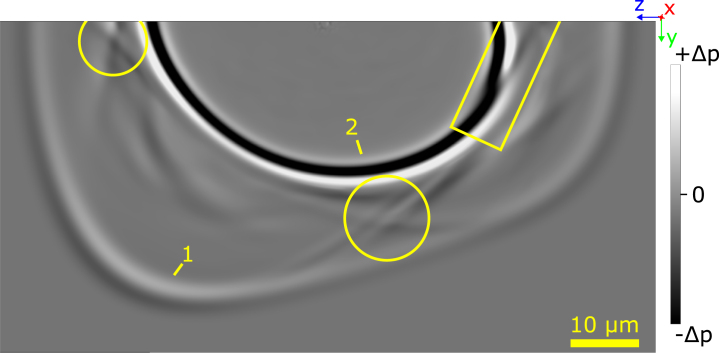
Snapshot of the simulated wave propagation in quartz. The grayscale displays the local pressure variations. HVPSAW (1) and SAW (2) modes can be observed in all directions. Rectangle marks overlap of SAW and PSAW modes due to close propagation velocity. Circles show folding of the PSAW mode with characteristic cusp shape. Material coordinate frame orientation is shown in the top right.

A circular measurement grid (see Fig. 2) with angle steps of 2.5∘ was used to determine angle-dependent propagation by tracking HVPSAW and SAW waves using an adaptive peak finding method. For a small angle range where SAW and PSAW are very close or overlap, no reliable wavefront tracing was possible (see Fig. 3). The material parameters used for the simulation are presented in Table 1 with the stiffness tensor C in Voigt notation and density ϱ. For anisotropic cases, the material coordinate system had to be rotated to match the desired orientation. In the case of x-cut quartz, the material x-axis was aligned with the model $x_{3}$-axis.

**Table 1:** Material parameters used in simulation.

Fused silica [44]	X-cut quartz [45]
C = −78−16−16−0−0−0−78−16−0−0−0−78−0−0−0−31−0−0sym.−31−0−31GPa	C = −87−7−13−18−0−0−87−13−18−0−0−106−0−0−0−58−0−0sym.−58−18−40GPa
$\varrho =2201\;kg\;m^{-3}$	$\varrho =2650\;kg\;m^{-3}$

## 4 Results and discussion

A snapshot of the simulated pressure change is shown in Fig. 3 for x-cut quartz. Since the excitation is in the out-of-plane direction, the in-plane dominant HVPSAW are considerably lower in amplitude than the out-of-plane dominant SAW mode. To display all modes simultaneously, Fig. 3 was composed using appropriate grayscale settings for each mode. Therefore, the gray values correspond to a local pressure change and are not absolute. The SAW and HVPSAW modes are clearly visible and show strong anisotropy. The overlap due to similar propagation velocities between SAW and PSAW is observed in a small angle range marked with a yellow rectangle. Its angular location agrees with measurements by Lewis and Patterson [41]. The PSAW mode can be observed in at least two areas marked with yellow circles in Fig. 3. Here, the slowness profile of the modes changes in curvature so that for a given direction, multiple velocity solutions exist, leading to a characteristic cusp shape also present in the $QT_{H}$ bulk mode at similar angular locations [30]. The other observed features could not be identified with sufficient confidence.

**Fig. 4:**
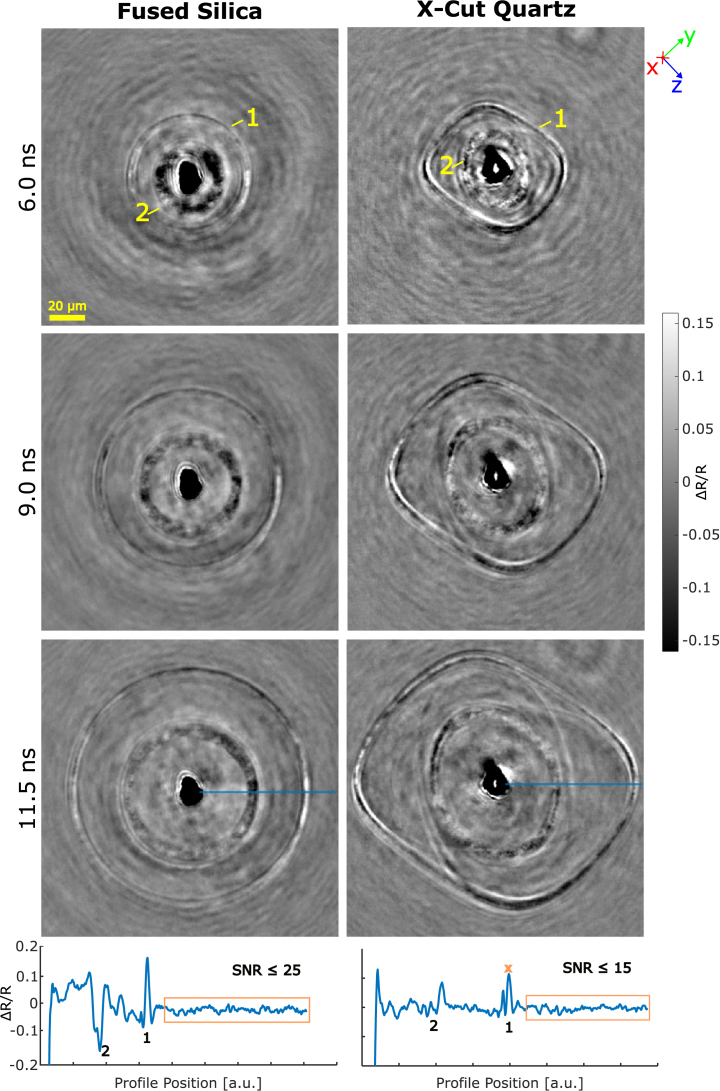
ΔR/R images at delay times from 6nsto11.5ns of fused silica (left) and quartz (right) with time progressing from top to bottom. Images are averages of ten measurements at each time delay. The scale denotes 20µm, 1 marks the HVPSAW and 2 the SAW wavefront position. Quartz material coordinate system is shown on the top right. The bottom row shows the ΔR/R profiles for each measurements along the blue line in the pictures at 11.5ns. Reference area for SNR calculation is marked by a box; x marks the signal value chosen for reference (HVPSAW peak). The reference area is not shown due to cropping of the images.

The ΔR/R images of the two measurements are shown in Fig. 4 for three selected probe beam time delays (6.0ns, 9.0ns and 11.5ns). To increase the SNR, ten measurements per time delay were averaged. In the Supplemental Materials section of this work, animated gifs of the full measurement are available, which provide a better view of the wavefront progression than stationary images. The ablated area is visible in the center of the images as a dark spot. Sharp lines show the current wavefront position. In both measurements, the HVPSAW and SAW modes could be identified at all sample angles. The maximum observed ΔR/R modulation is slightly below 0.2. The average peak heights are around $\Delta R/R_{av}=0.05$. With noise standard deviation $\sigma_{N}=7.4\times 10^{-3}$ this gives an average SNR of

(7) $$SNR_{av}=\frac{\Delta R/R_{av}}{\sigma_{N}}=6.8\;.$$

The Supplemental Material Section contains a comparison with unaveraged measurements. The pump beam split-off for the birefringent quartz can be observed as a second focal point in the upper right of the images in Fig. 4. However, the energy of this second excitation is low enough that no influence on the measured wavefronts is observed. The bottom of Fig. 4 shows example ΔR/R profiles along selected directions for both measurements including SNR values.

In the case of fused silica, both the HVPSAW and SAW modes show circular propagation as a result of the material isotropy. The amplitude of the observed reflectivity peaks is not symmetric as expected for isotropic materials but shows a noticeable angular dependence which remains constant across time delays. The setup was checked with respect to misalignment or influences on the observations, but a symmetric sensitivity could not be achieved. An explanation for this observation could be strain-induced birefringence caused by the propagating mechanical wavefronts. This was also observed by Yamazaki et al. [20]. Further investigation of this effect with a polarization-sensitive setup is planned.

The frequency bandwidth was estimated by measuring the full width at half-maximum (FWHM) of the HVPSAW in fused silica. By analyzing the intensity profile along the propagation of the pulse, the FWHM was measured at around 2µm. Assuming a Gaussian pulse shape with a time-bandwidth product of 0.44 and a propagation velocity of $6000\;m\;s^{-1}$ (see Section 3.2) this gives a frequency bandwidth of around 1.3GHz.

**Fig. 5:**
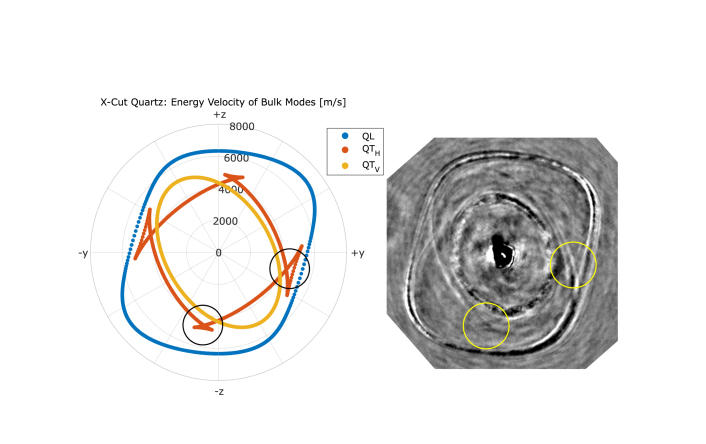
Left: Calculated energy velocity of bulk modes in x-cut quartz (left) and snapshot of the wave propagation in x-cut quartz at 10.7ns (right). The axes show the crystal orientation and the image has been rotated to match the orientation. The QL mode shape matches the observations of the HVPSAW in simulation and experiment. The folding of the QT_H_ is visible in the same direction as for the PSAW in the experiment (black/yellow circles). (For interpretation of the references to color in this figure legend, the reader is referred to the web version of this article.)

For quartz, both HVPSAW and SAW modes were observable. The overlap of SAW and PSAW (yellow rectangle in Fig. 3) cannot be distinguished. A characteristic cusp shape of the PSAW can be observed especially for longer probe beam delays. Fig. 5 shows the calculated energy velocity for the bulk modes of x-cut quartz. The calculations were performed using the *christoffel* Python toolset [39] without the piezoelectric effect. Fig. 5 (right) shows the measurement of quartz at 10.7ns and has been rotated so that the material frame matched the calculated plot to the left. The position of the folded QT_H_ mode (black circle) agrees well with the observation of the PSAW (yellow circle) and the simulations (Fig. 3). The second folded region at ±z is considerably weaker, but appears in the simulations in the same direction as observed in the measurement. This shows that the PSAW is related to the $QT_{H}$ bulk mode, as can be shown with the method of partial waves [31].

**Fig. 6:**
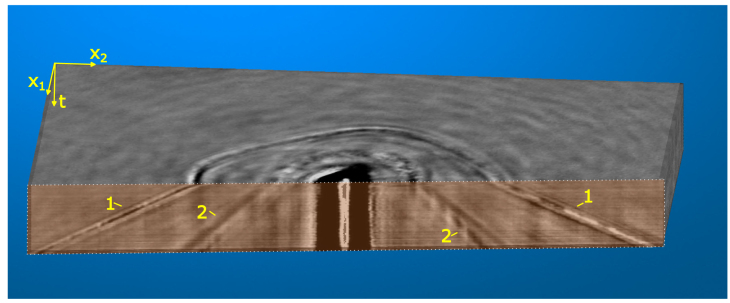
Image stack of x-cut quartz measurement with time delay progressing from top (6ns) to bottom (11.5ns). Wave propagation of the HVPSAW (1) and the SAW (2) is visible in the cross-sectional view (orange tint).

Fig. 6 shows a cross sectional slice through the entire quartz dataset. The measurement is displayed as an image stack with time progressing from top (6ns) to bottom (11.5ns). Propagating wave modes can be identified as linear streaks that are directed outward from the image center. Nonlinear acoustic effects, as observed by Pezeril et al. [24] for the arrival of a Gaussian pulse at an interface, could not be detected for the observed surface wave modes. The wavefronts show no curvature or dispersion, suggesting a propagation in the interface layer, orthogonal to the camera plane and without interaction with additional boundaries. The measured velocities are in agreement with elastic wave propagation.

Even in the thermoelastic excitation regime, SAWs can show nonlinear behavior [46]. Since femtosecond laser ablation is a strongly nonlinear process [47], [48] where pressure amplitudes up to the TPa regime in bulk dielectric media are predicted [49], [50], the question arises of why such effects are not observable in this work. The simulation of ultrashort light-matter interaction is still a matter of current research with elastodynamic [51], molecular dynamic [52] or hydrodynamic [53] approaches all showing excellent results. To calculate the expected initial pressure amplitudes, a one-dimensional two-temperature hydrodynamic simulation [53], [54] was used to simulate the ablation of the aluminum layer on fused silica substrate for the laser parameters used in this work. The Supplemental Materials section contains more detailed information regarding the simulation methodology as well as a plot of the pressure distribution over time.

For a fluence of $1.4\;J\;{cm}^{-2}$, the simulation predicts a maximum pressure in the Al thin-film ablation layer of around 80GPa which induces 50GPa in the transmission boundary layer of fused silica a few picoseconds after excitation. This is well in the nonlinear regime [46]; therefore, shock wave formation is expected to occur during ablation within the picosecond timescale. However, when the simulation is extended to 1ns, the maximum pressure is reduced to around 2GPa due to the strong attenuation of the propagating shock front [55] even without considering geometric damping effects. This explains the absence of (observable) nonlinearities in the experiments, since the probe delay times are at least 6ns in this work. At this time, the pressure amplitude is expected to be even further attenuated.

A common condition for the validity of the elastic wave assumption, using c as the speed of sound, is $\left|p_{max}\right|\ll \rho \;c^{2}$ [56]. In the case of fused silica, this is equivalent to $\left|p_{max}\right|\ll 78\;GPa$ suggesting linear behavior for sub-GPa pressure amplitudes. In 3D, an increased attenuation of the pressure amplitude is expected, since the energy is shared between multiple wave modes that cannot be modeled within the one-dimensional framework. For further research, the observation of the transition of the excited shock waves in the interface layer from a strong nonlinear behavior to the elastic regime within sub-ns time frames could be of interest [57]. Here, the comparative simulation of wave propagation will be considerably more challenging than in the present work. Since the nonlinear effects are dependent on the bandwidth and amplitude of the excitation [46], the simulation parameters must closely match the experimental ones to produce usable results.

**Fig. 7:**
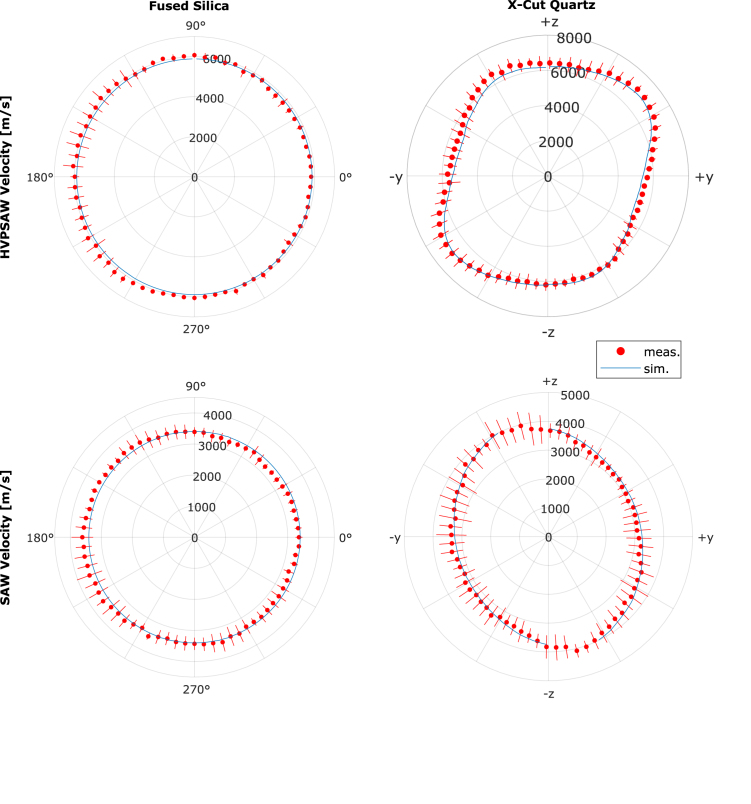
Velocities of HVPSAW and SAW modes observed in measurement (red) and simulation (blue) for fused silica and x-cut quartz. Error bars show the 95% confidence interval of the linear regression of the measurement. For quartz, the coordinates are aligned to the material frame. (For interpretation of the references to color in this figure legend, the reader is referred to the web version of this article.)

The results of the measured wave velocities are shown in Fig. 7 (red marker with error bar), compared to the simulation (blue lines). The error bars display the 95% confidence intervals of the fitted points. For each angle, six wavefront positions are recorded every 5∘, resulting in 432 measurements per wave mode. The predicted angle dependence of wave propagation is clearly reproduced for both materials. In the case of fused silica, the velocities were $c_{SAW}=3411\left(75\right)\;m\;s^{-1}$ for the SAW mode and $c_{HVPSAW}=5955\left(94\right)\;m\;s^{-1}$ for the HVPSAW mode, averaged over all angles. Using both velocities and assuming $c_{HVPSAW}\approx c_{L}$, the Young’s modulus E and Poisson’s ratio ν of fused silica are estimated. Solving the relation [35]

(8) $$c_{L}=\sqrt{\frac{E}{\rho }\;\frac{1-\nu }{\left(1+\nu \right)\;\left(1-2\nu \right)}}$$

(9) $$c_{R}=\frac{0.87+1.12\nu }{1+\nu }\;\sqrt{\frac{E}{\rho }\;\frac{1}{2\left(1+\nu \right)}}$$

for E and ν and assuming a density $\rho =2201\;kg\;m^{-3}$ results in values within 2% of the literature values (Table 2).

The SAW mode of x-cut quartz ranges from $3027\;m\;s^{-1}\;\text{to}\;4080\;m\;s^{-1}$ and the HVPSAW mode from $5389\;m\;s^{-1}\;\text{to}\;7093\;m\;s^{-1}$. The gaps in the simulated SAW velocities for quartz are the result of an overlap with the PSAW mode that hinders the detection of the peak (see Fig. 3). Due to the anisotropy of the propagation in quartz, solving the inverse problem using the measurement data to determine the stiffness tensor is considerably more challenging. However, the data could be used as reference within iterative model-based approaches in further works. Stoklasová et al. [58] demonstrated this by employing the Ritz-Rayleigh method to transform the nonlinear system (3) into a linear eigenvalue problem, which allows fast calculation of SAW as well as bulk velocities of all material symmetry classes and surface orientations. By optimizing the objective function defined as the difference between the calculated and measured velocities, they were able to determine the elastic constants of two InP wafer cuts within 1percent. It is notable that the HVPSAW and SAW velocity data – which we can both detect in our setup – is required for precise determination of all stiffness constants. Using only SAW data leads to high discrepancies for the stiffness constants that are coupled with the longitudinal mode. Alternative numerical methods to use as reference for optimization can be found in e.g. [59].

**Table 2:** Results for parameter estimation for fused silica. Measured and literature values [44] for E and ν with individual errors.

	E	ν
Meas.	72.8GPa	0.167
Lit.	72.5GPa	0.170
Error	0.5%	−1.9%

Table 3 shows the absolute and relative deviations between measurement and simulation. The mean relative error lies between 2.1% for the HVPSAW in fused silica and 5.3% for the HVPSAW in quartz.

The results show a good agreement between measurement and simulation. The measured and simulated propagation velocities overlap mainly within the 95% confidence interval of the measurement. For the anisotropic quartz sample, the theoretical angle dependence of both modes is reproduced in the measurements.

**Table 3:** Comparison of measurement error relative to the simulation, averaged over all angles. Relative errors $\left|\frac{\Delta v}{v_{Sim}}\right|$, absolute errors |Δv| and standard deviation σ of the measured velocities compared to the simulated data.

Sample	Wave mode	$\left|\frac{\Delta v}{v_{Sim}}\right|$ [%]	|Δv| [$m\;s^{-1}$]	σ [$m\;s^{-1}$]
Fused silica	HVPSAW	2.1	123.7	75.4
	SAW	2.8	94.0	54.2
X-cut quartz	HVPSAW	5.3	324.1	241.1
	SAW	2.3	79.4	45.6

Compared to existing interferometric and photodiode-based methods, which work with sub-nanojoule pulse energies (≈0.5nJ [21], [22]) in the photoelastic regime, the required energies in our work are several orders of magnitude higher (4.6µJ). Due to the necessity of moving the sample between shots, the crystal orientation or stiffness parameters have to be constant across a few centimeters. The fluence is within the ablation regime of the aluminum layer but not of the fused silica/quartz substrate. Therefore, the measurement is destructive, although the damaged area is small. The speed is increased by a factor of ten (≤40s for a 10x averaged image) compared to point-based detection methods [15], [18]. This can be further increased by reducing number of averages at the cost of SNR (see Supplemental Materials section). The reflectometric measurement method is sensitive to the stress state of the material, allowing full-field waveform measurement including the detection of in-plane dominant modes for which interferometric measurement setups have low sensitivity. Using ten averages, the SNR is sufficient to consistently detect wave propagation in all directions for quantitative velocity measurements. The optical setup works in reflection measurement mode with a common pump–probe path. This allows for a relatively simple setup and requires only access to one side of the sample.

The optimal optical detection of surface waves depends, among others, on the desired frequency range, temporal resolution, measurement time and optical parameters of samples. Several specialized measurement techniques are available that are described in detail in a current review by Spytek et al. [60]. High sensitivities can be achieved for interferometric techniques with very low excitation energies. In the GHz range, Sagnac interferometers have been used with an SNR high enough to detect the HVPSAW by its small out-of-plane component [18], [61]. The photoelastic reflectivity change of a surface as well as the stress state in transparent media can be revealed with photodetectors with high sensitivities when combined with lock-in amplification of the signal [20], [22]. Fast full-field imaging with picosecond temporal resolution can be achieved with pump–probe microscopy by using detection with camera sensors at the cost of reduced sensitivity. When ablative excitation is allowed, this technique can visualize the propagation of surface waves without averaging. For lower energies, Pezeril et al. [24] demonstrated nondestructive measurement of a picosecond acoustic pulse after propagation through a thin gold layer. The excitation was achieved in the nondestructive regime by using a cobalt layer as impedance matched opto-acoustic transducer for high energy absorption and averaging of 1000 synchronized images. This sample design could be of interest for the experimental setup presented in this work, since currently a significant portion of the pump laser energy is reflected by the Al-layer when leaving the ablative regime, which further reduces the surface wave excitation strength. Other possibilities for improved excitation amplitude include engineering thin-film stacks so that a change in partial reflectance gives a large degree of contrast or using pump pulse wavelengths where the absorption of the sample is high, which could enable measurements without using coating layers. Furthermore, if the sensitivity can be enhanced, permitting ablation-free measurements, translation of the sample between successive shots can be avoided, enabling the detection of local variations in elastic properties.

## 5 Conclusion

In this work, we presented a measurement and imaging setup for multimodal elastic surface waves in transparent media based on laser pump–probe microscopy. Using subpicosecond laser pulses, wavefronts with frequency bandwidths in the gigahertz regime were excited, visualized, and quantitatively analyzed on fused silica and x-cut quartz wafers. The measurements were compared using finite element simulation models of wave propagation and showed good agreement within a few percent. Since the wave field was imaged at more than 50 delay times, full wave field movies could be created, showing the propagation of multiple surface wave modes.

The measurement speed is increased by two orders of magnitude compared to other reported techniques, whereas the sensitivity is lower than in the case of pointwise measurements using interferometric or photodiode-based techniques. This fast and full-field imaging of GHz acoustic waves can be of interest for quantitative material characterization of optical crystals or semiconductor wafers which have applications in surface acoustic wave filter devices. The goal of future research work is to improve the sensitivity, without compromising the measurement speed. A reduction in the excitation pulse energy below the ablation threshold could enable nondestructive characterization of the elastic parameters of the materials.

## CRediT authorship contribution statement

**Ruben Burger:** Writing – original draft, Visualization, Validation, Software, Methodology. **Goran E. Hallum:** Writing – original draft, Validation, Methodology, Investigation. **Ramon Auer:** Writing – review & editing, Validation, Investigation. **Dennis Schweiger:** Writing – review & editing, Visualization, Validation, Software, Investigation. **David Redka:** Conceptualization, Investigation, Validation, Visualization, Writing – review & editing. **Matthias Domke:** Writing – review & editing, Investigation. **Christian U. Grosse:** Writing – review & editing, Supervision. **Heinz P. Huber:** Writing – review & editing, Resources, Funding acquisition, Conceptualization. **Datong Wu:** Writing – review & editing, Project administration, Conceptualization, Supervision.

## Declaration of competing interest

The authors declare the following financial interests/personal relationships which may be considered as potential competing interests: Ruben Burger reports article publishing charges was provided by German Research Foundation. Heinz P. Huber reports financial support was provided by German Research Foundation. If there are other authors, they declare that they have no known competing financial interests or personal relationships that could have appeared to influence the work reported in this paper.

## Data Availability

Data will be made available on request.

## Appendix A Supplementary data

Supplemental to this manuscript, two video sequences of wavefront propagation in both materials are included in the submission as *FusedSilicaGray.gif* and *QuartzGray.gif*. Both are available online as well as a document containing four additional figures and information regarding the ablation simulation.

MMC S1 The Suppmentary document contains four additional figures and further details regarding the ablation simulation.
