# Supplementary material for: Velocimetry of GHz elastic surface waves in quartz and fused silica based on full-field imaging of pump–probe reflectometry
Source: Photoacoustics. 2024 Jun 11;38:100627. doi: 10.1016/j.pacs.2024.100627 (PMC11225356; doi:10.1016/j.pacs.2024.100627)
Supplement: MMC S1 — The Suppmentary document contains four additional figures and further details regarding the ablation simulation. [file mmc1.pdf]

Supplemental Material: Velocimetry of GHz Elastic Surface Waves in Quartz and Fused Silica based on Full-Field Imaging of Pump-Probe Reflectometry

Ruben Burger[a,b,*], Goran E. Hallum[a], Ramon Auer[a], Dennis Schweiger[a], David Redka[a], Matthias Domke[c], Christian U. Grosse[b], Heinz P. Huber[a], Datong Wu[a]

[a]*Munich University of Applied Sciences HM, Lothstr. 34, Munich, 80335, Bavaria, Germany*
[b]*Technical University of Munich - Chair of Nondestructive Testing, Franz-Langinger-Str. 10, Munich, 81245, Bavaria, Germany*
[c]*Vorarlberg University of Applied Sciences - Josef Ressel Center for Material Processing with Ultrashort Pulsed Lasers, Feldgut 9, Rankweil, 6830, Vorarlberg, Austria*

Abstract

Supplemental Materials to Paper "Velocimetry of GHz Elastic Surface Waves in Quartz and Fused Silica based on Full-Field Imaging of Pump-Probe Reflectometry".

1. Additional Figures

Figure 1 shows the comparison between unaveraged and averaged image quality for x-cut quartz at delay times of 11.5 ns. The SNR scales with the square root of the number of averages, leading to 3× improvements in our case which can be observed for the measurements along the line profiles at the bottom of Figure 1.

*Corresponding author
Email address: ruben.burger0@hm.edu (Ruben Burger)

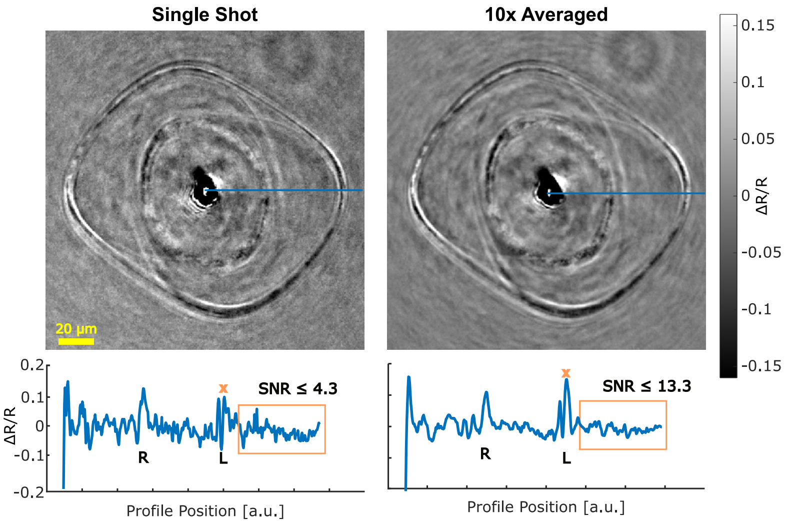

Figure 1: Comparison of single measurement (left) and 10x averaged (right).

Figure 2 shows the measurement software and data analysis. The red crosses show the detected wavefront positions for the HVPSAW at one delay time. At the bottom, a line profile along the selected angles is displayed.

Figure 3 shows the calculated piezoelectric coupling K for x-cut quartz. The maximum value is reached slightly offset from the material y-axis and the minimum is along the z-axis.

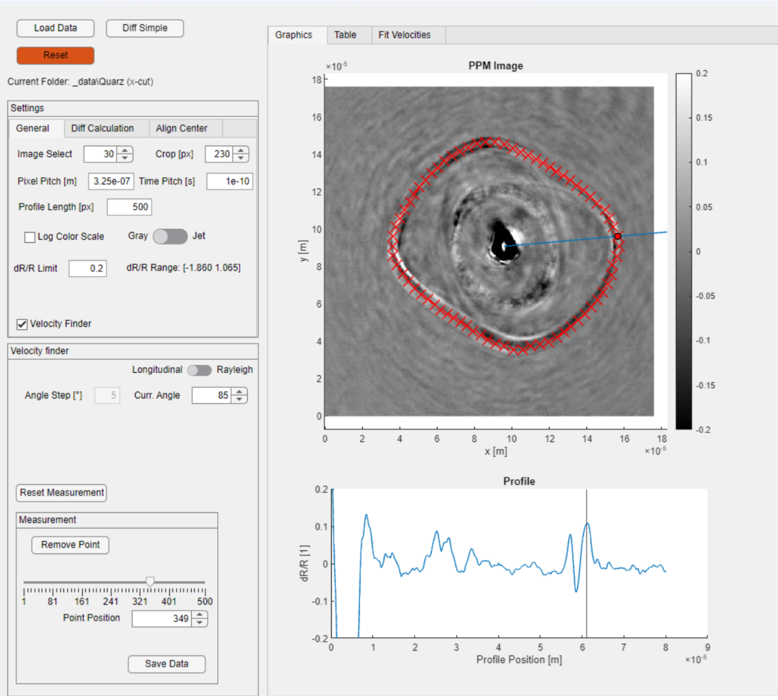

Figure 2: Matlab interface for quantitative velocity measurement.

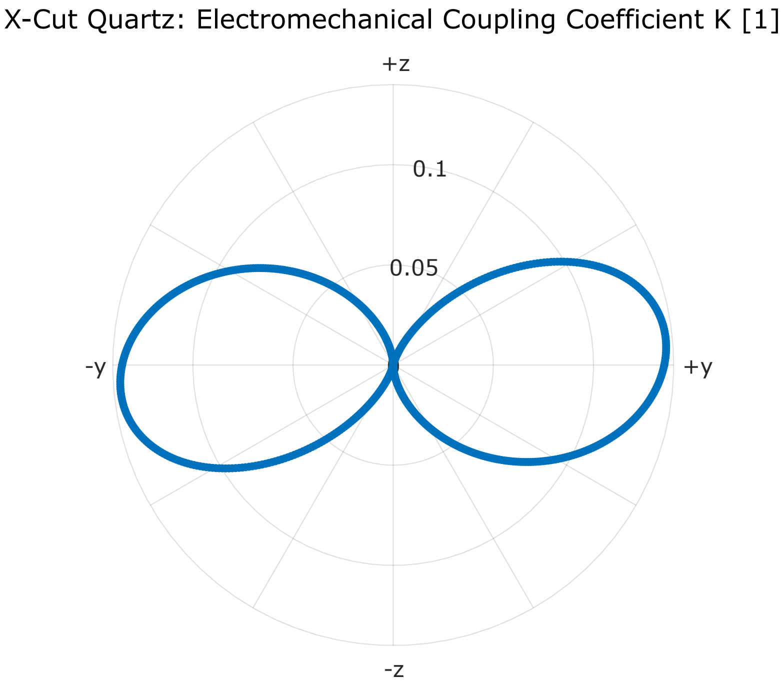

Figure 3: Piezoelectric coupling coefficient K of x-cut quartz.

2. Details of ablation simulation

Ultrafast laser ablation simulations were performed with one-dimensional two-temperature hydrodynamic code developed by Povarnitsyn et al. [1]. For our simulations, we employed a layered system consisting of Al on SiO_2 in the form of fused silica. In addition to the transport and optical parameters of Al related to the two-temperature model (TTM) [2, 3], the material response to ultrafast laser excitation is modeled within this framework by an equation of state (EOS). For Al, a two-temperature wide-range multi-phase EOS was utilized, developed by Khishchenko [4]. For fused silica, an implemented simple thermal two-temperature EOS model based on the quasi-harmonic Debye model was used [5]. This model is calibrated primarily using shock-wave data (shock Hugoniots, release isentropes, and sound speed measurements behind the shock front). The ideal Fermi-gas model was used for the electronic term.

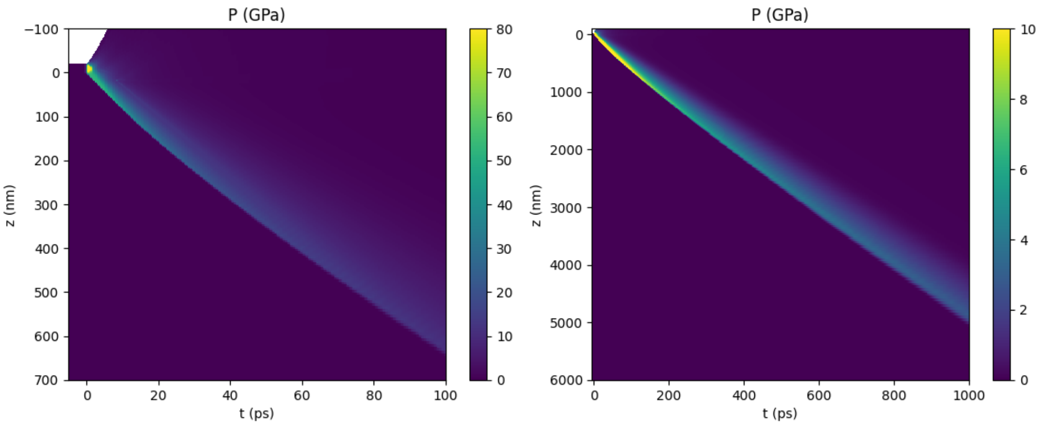

Figure 4: Simulation of the generated pressure during ablative excitation within 100 ps (left) and up to 1 ns (right). Note the different color scale.

The simulation was performed with a 20 nm Al film deposited on a 10 µm fused silica substrate. A finite difference element size of 2 nm was used, increasing to 10 nm within the SiO_2 substrate for depths exceeding 1 µm. Substrate side laser irradiation was simulated with a fluence of $1.4\,\mathrm{J\,cm^{-2}}$, wavelength of 1056 nm, and pulse duration of 0.7 ps. It should be noted that the SiO_2 substrate within the simulation does not interact with the laser pulse. Time delays up to 1 ns were investigated, with a particular focus on the ablation induced pressure within the SiO_2 substrate. We emphasize that the chosen method for simulating thin-film ultrafast laser ablation has been extensively validated, as demonstrated e.g. by Olbrich et al. [6] for thin gold films. The main limitation of the 1D simulation compared to experiments is that it may overestimate the pressure induced in the SiO_2 substrate, as already described in the main manuscript. This is because the 1D model does not account for 3D losses, which would tend to reduce the pressure buildup.

The pressure distribution over time is shown in Figure 4 for up to 100 ps (left) and 1 ns (right) after excitation. The temporal development of the pressure distribution after excitation at $t = 0\,\mathrm{ps}$ is displayed. The interface between Al and fused silica is at $z = 0\,\mathrm{nm}$ with the Al layer extending to $z = -20\,\mathrm{nm}$ and the fused silica for $z > 0\,\mathrm{nm}$. The small yellow section in Figure 4 (left) shows the high pressures created during the ablation of the Al layer directly after excitation. The pressure pulse transmitted in the fused silica shows significant curvature, especially during the first 100 ps, indicating amplitude-dependent propagation velocities of the shock wave. Figure 4 (right) shows the high attenuation of the pulse after 1 ns with pressure amplitudes reduced to $\approx 2\,\mathrm{GPa}$. Note the different pressure scales of the

colormaps.

References

[1] M. E. Povarnitsyn, T. E. Itina, M. Sentis, K. Khishchenko, P. Levashov, Material decomposition mechanisms in femtosecond laser interactions with metals, Physical Review B 75 (2007) 235414.

[2] M. E. Povarnitsyn, N. E. Andreev, E. M. Apfelbaum, T. E. Itina, K. V. Khishchenko, O. F. Kostenko, P. R. Levashov, M. E. Veysman, A wide-range model for simulation of pump-probe experiments with metals, Applied Surface Science 258 (2012) 9480–9483.

[3] J. Winter, D. Redka, J. Minár, M. Schmidt, H. P. Huber, Resolving transient temperature and density during ultrafast laser ablation of aluminum, Applied Physics A 129 (2023) 665.

[4] K. Khishchenko, Equation of state and phase diagram of tin at high pressures, in: Journal of Physics: Conference Series, volume 121, IOP Publishing, 2008, p. 022025.

[5] K. V. Khishchenko, Temperature and heat capacity of polymethyl methacrylate behind the front of strong shock waves, Teplofizika vysokikh temperatur 35 (1997) 1002–1005.

[6] M. Olbrich, T. Pflug, C. Wüstefeld, M. Motylenko, S. Sandfeld, D. Rafaja, A. Horn, Hydrodynamic modeling and time-resolved imaging reflectometry of the ultrafast laser-induced ablation of a thin gold film, Optics and Lasers in Engineering 129 (2020) 106067.
